# Periodontal and peri-implant status and whole salivary interleukin 1-beta levels among individuals using selective serotonin reuptake inhibitors: an observational study

**DOI:** 10.1186/s12903-023-02908-0

**Published:** 2023-05-22

**Authors:** Shatha Subhi ALHarthi, Munerah S. BinShabaib, Abdulrahman Alwahibi, Shahinaz Gamal, Eldin Elashiry, Sarah E. Almershed, Haifa Abdulrahman Alkhamis, Lamyia Anweigi

**Affiliations:** 1 Department of Preventive Dental Sciences, College of Dentistry, Princess Nourah Bint Abdulrahman University, Riyadh, Saudi Arabia; 2 Department of Psychiatry, College of Medicine, King Saud University Medical City, King Saud University, Riyadh, Saudi Arabia; 3 Department of Oral Medicine, Periodontology, Oral Diagnosis and Oral Radiology, Faculty of Oral and Dental Medicine, Misr International University, Cairo, Egypt; 4 Specialist in Periodontics, Ministry of Health, Kuwait City, Kuwait; 5 Lecturer and Examiner at the Kuwait Board of Advanced General Dentistry, Kuwait University, Kuwait City, Kuwait; 6 Faculty of Dentistry, Kuwait University, Kuwait City, Kuwait; 7 Staff Dentist at National Guard Health Affairs (NGHA), Riyadh, Saudi Arabia; 8 College of Dental Medicine, QU Health, Qatar University, Doha, Qatar

**Keywords:** Alveolar bone loss, Attachment loss, Depression, Periodontal, Peri-implant diseases, Probing depth, Saliva

## Abstract

**Objective:**

Selective serotonin reuptake inhibitors (SSRI) are commonly used for managing psychological diseases such as depression. These disorders are also directly associated with periodontal and peri-implant diseases, namely periodontitis and peri-implantitis, respectively. It is hypothesized that there is no difference in periodontal and peri-implant clinicoradiographic status and unstimulated whole salivary interleukin (IL)-1β levels in participants using selective serotonin reuptake inhibitors (SSRI) and controls (individuals not using SSRI). The aim of the present observational case-control study was to compare periodontal and peri-implant clinicoradiographic statuses and whole salivary IL-1β in participants using SSRI and controls.

**Methods:**

Users of SSRI and controls were included. In all participants, periodontal (plaque index [PI], gingival index [GI], probing depth [PD], clinical attachment loss [AL] and marginal bone loss [MBL]) and peri-implant (modified PI [mPI], modified GI [mGI], PD and crestal bone loss [CBL]) were assessed. Unstimulated whole saliva was collected and IL-1β levels were determined. Information related to duration of implants in function, duration of depressive symptoms and treatment of depression was retrieved from healthcare records. Sample-size was estimated using 5% error and group comparisons were performed. P < 0.05 was considered statistically significant.

**Results:**

Thirty-seven SSRI users and 35 controls were assessed. Individuals using SSRI had a history of depression of 4.2 ± 2.5 years. The mean age of SSRI-users and controls were 48.7 ± 5.7 and 45.3 ± 5.1 years, respectively. Tooth brushing twice daily was reported by 75.7% and 62.9% SSRI-users and controls, respectively. There was no statistically significant difference in PI, mPI, GI, mGI, PD, clinical AL, numbers of MT and mesial and distal MBL and CBL among individuals using SSRI compared with controls (Tables 3 and 4). The unstimulated whole salivary flow rate in individuals using SSRI and controls was 0.11 ± 0.003 and 0.12 ± 0.001 ml/min, respectively. Whole salivary IL-1β levels in individuals using SSRI and controls were 57.6 ± 11.6 pg/ml and 34.6 ± 5.2 pg/ml, respectively.

**Conclusion:**

Users of SSRI and controls demonstrate healthy periodontal and peri-implant tissue statuses with no marked differences in whole salivary IL-1β levels provided oral hygiene is stringently maintained.

## Introduction

Mood disorders (MD), such as depression are a global crisis [1]. Moreover, the corona virus disease-19 pandemic has exacerbated the prevalence of anxiety and depressive disorders worldwide [2]. According to a recent study [2], there are nearly 3153 individuals with major depressive disorders per 100,000 population globally. Participants with MD including depression often isolate themselves and neglect daily routine activities such as regular intake of meals and hygiene maintenance including oral hygiene maintenance (OHM) [3, 4]. It has been reported that oral diseases such as dental caries (DC) and periodontitis are more often manifested in individuals with than without depression [3]. It has been reported that the prevalence of periodontitis in individuals with MD is approximately 57% [5]. With regards to dental implants, the precis prevalence of peri-implant diseases in individuals with MD remains unclear, it has been reported that individuals using antidepressants are more susceptible to implant failure in contrast with controls (individuals not using antidepressants) [6, 7].

The selective serotonin reuptake inhibitors (SSRI) inhibit the serotonin transporter at the presynaptic axon terminal, which sequentially increases the amount of serotonin remains in the synaptic cleft [8]. This stimulates postsynaptic receptors for an extended period.[8] In comparison with other antidepressants such as mono-amine oxidase inhibitors (MOI) and tricyclic antidepressants (TCA), SSRI have due to fewer effects on adrenergic, cholinergic, and histaminergic receptors [9]. Fluoxetine belongs to the family of SSRI and is commonly used for the management of patients with MD [10]. Fluoxetine is well-absorbed after oral ingestion, exhibits a non-linear pharmacokinetic profile and has an elimination half-life of 7–15 days [11]. According to Altamura et al. [11] there is no association between advancing age and pharmacokinetics of Fluoxetine. However, Fluoxetine should be cautiously administered to patients with impaired metabolic activity [11]. Results from studies [12–14] on animal-models have shown that fluoxetine possesses anti-inflammatory properties as it reduces the production of destructive inflammatory cytokines and prostaglandin E_2_. Branco-de-Almeida and co-workers [12] showed that fluoxetine administration minimizes the risk of alveolar bone loss (ABL) in rats with ligature induced periodontitis. Results from a study [15] on mice showed that fluoxetine administration augments trabecular bone formation in mice. Furthermore, in a systematic review of studies on animal-models Muniz et al. [16] proposed that anti-depressants including SSRI (fluoxetine) can help in the management of periodontal disease via their anti-inflammatory properties. However, clinical results by Bey et al. [17] showed that participants using SSRI had significantly higher scores of clinical attachment loss (AL), probing depth (PD), plaque index (PI) and gingival index (GI) compared with individuals not using SSRI. With regards to clinical implant dentistry, studies [6, 7, 18–21] have shown that the risk of implant failure is high in users of SSRI compared with controls; however, it is notable that that a prior sample-size estimation (power analysis) was not performed in most of them [6, 7, 18–20]. Therefore, from the authors’ perspective, such results should be cautiously interpreted. Unstimulated whole saliva (UWS) is a complex biologic fluid that expresses raised levels of proinflammatory cytokines such as interleukin 1 beta (IL-1β) under periodontal and peri-implant inflammatory conditions such as periodontitis and peri-implant mucositis and peri-implantitis, respectively [22, 23]. Since SSRI such as fluoxetine possess anti-inflammatory properties [12, 14]; it is hypothesized that there is no difference in periodontal and peri-implant clinicoradiographic status and unstimulated whole salivary IL-1β in individuals using SSRI and controls.

The aim of the present power-adjusted case-control study was to compare the periodontal and peri-implant clinicoradiographic status and unstimulated whole salivary IL-1β in participants using SSRI and controls.

## Materials and methods

### Ethics statement

The current study was performed in accordance with the guidelines of the Helsinki declaration as revised in the year 2013. Participation was completely voluntary and all participants were mandated to read and sign a written informed consent form. Ethical approval was obtained from the ethics committee of Centre for specialist dental practice and clinical research, Riyadh, Saudi Arabia (UDCRC/025 − 16).

### Study design, location and timing

The present observational case-control study was performed at the Centre for specialist dental practice and clinical research, Riyadh, Saudi Arabia between September 2021 and June 2022.

### Inclusion and exclusion criteria

Individuals with the following characteristics were considered eligible for inclusion: (a) medically diagnosed depression as the ICD-10 symptom rating (ISR) [24] provided in medical records; (b) participants with at least one missing tooth replaced with dental implant in either jaw; (c) participants using SSRI; (d) self-reported medically healthy individuals. The exclusion criteria were as follows: (a) refusal to signing the consent form; (b) alcohol usage; (c) tobacco-smoking and use of smokeless tobacco; (d) surgical and/or non-surgical periodontal/peri-implant treatment within 6-months; (e) use of antibiotics, steroids, non-steroidal anti-inflammatory drugs, bisphosphonates within the past 90 days; (f) participants with hypertension, cardiovascular disease renal disorders, hepatic disorders and viral infections such as HIV/AIDS and (g) presence of third molars, and grossly carious and supernumerary dentition.

### Assessment of patient’s medical and dental records

Data pertaining to patient age, gender, education status, duration since diagnosis of depression, treatment of depression, dosage of medications, duration of implants in function, number of implants placed per individual, implant jaw location, implant dimensions, implant surface characteristics, depth of implant insertion (subcrestal or bone level), mode of prostheses retention (cement or screw), implant insertion torque and implant abutment connection was retrieved from participants’ medical and dental records. These records were assessed by the principal investigator.

### Classification of participants’ education status

Individuals that reported to have attained education up to the 10th grade were classified as having “School-level education” [25, 26]. Individuals that had attended two-years of college after completing school education were classified as having “College-level education” [26]; and individuals that reported to have graduated from a university after completion of college-level education were classified as having “University-level education” [26, 27].

### Invitation for participation

Invitation letters that explained the purpose and methodology of the present study in simple English and Arabic were dispatched to 63 individuals with depression and 59 controls. A consent form was also sent along with the invitation letter and individuals that agreed to participate in the present investigation were requested to visit the Centre for specialist dental practice and clinical research, Riyadh, Saudi Arabia for a clinical and radiological examination. All individuals were informed that personal information including but not limited to their name, age, address, contact details, and gender will be kept strictly confidential; and that there are no financial benefits or consequences associated with participation and withdrawal, respectively in regards to the present study.

### Periodontal and peri-implant clinicoradiographic parameters

Full-mouth plaque and gingival indices (PI [28] and GI [29]) were recorded on 4-sites (distal, palatal/lingual, mesial and buccal/facial) per tooth. Clinical AL [30] and PD [31] were assessed on six sites (mesiobuccal, midbuccal and distobuccal, mesiolingual/palatal, midlingual/palatal and distolingual/palatal) per tooth. Clinical AL and PD were recorded in millimeters (mm). Peri-implant modified plaque and gingival indices (mPI [28] and mGI [29], respectively) were recorded on 4-sites (distal, palatal/lingual, mesial and buccal/facial) per implant. In all participants PD was recorded on six sites per implant using a plastic graded probe (UNC12, Colorvue® plastic probe, Hu-Friedy, Chicago, IL). Full mouth digital bitewing radiographs were taken to assess the crestal and marginal bone loss (MBL) around implants and teeth, respectively. The crestal bone loss (CBL) and MBL were measured as linear distances from two mm below the implant abutment connection and cement-enamel junction, respectively to the alveolar crest [32, 33].

### Collection of unstimulated whole saliva and assessment of interleukin 1-beta levels

The UWS samples were collected during 8:00 am and 9:00 am with individuals being in a fasting state as described elsewhere [34, 35]. Participants were seated in a quiet room and requested to allow saliva to accumulate in the mouth for 5 min [36, 37]. At the end of this duration participants were requested to expectorate the accumulated saliva into a measuring cylinder through a disposable plastic funnel. The amount of saliva collected was recorded and the whole salivary flow rate was determined in millimeters in minutes (ml/min). The collected saliva samples were centrifuged at 3000 x g for 15 min in a cold room. The supernatant was collected and stored in sterile plastic tubes with lid and stored at -70 degrees Celsius. All samples were assessed for IL-1β levels within 48 h using commercially available sandwich enzyme linked immunosorbent assay kits (SALIMETRICS, Catalog # 1-3902, PA 16,803). The kits were used according to manufacturers’ instructions (assay range: 3.13–200 pg/ml).

### Sample-size estimation and statistical analyses

Sample-size estimation was done using data from a pilot investigation. A computer-based software was used for power analysis (G^*^Power 3.1.9.2, Bonn Germany). It was projected that at least 33 and 33 SSRI-users and controls, respectively are needed to detect a 2 mm difference in periodontal and peri-implant PD between the groups. With this sample-size, the study was project to have a power of 80% with an alpha of 5%. Quantitative assessment was performed using the SPSS software (version 24.0, IBM Corp., Statistical solutions, Chicago, IL, USA). Data normality was assessed using the Shapiro-Wilk test. Periodontal and peri-implant clinico-radiographic parameters were compared using the Mann-Whitney U-test. Correlation between demographic and periodontal and peri-implant parameters with whole salivary IL-1β was assessed using logistic regression models. Level of significance was set at P < 5%.

## Results

### Demographics

**Fig. 1:**
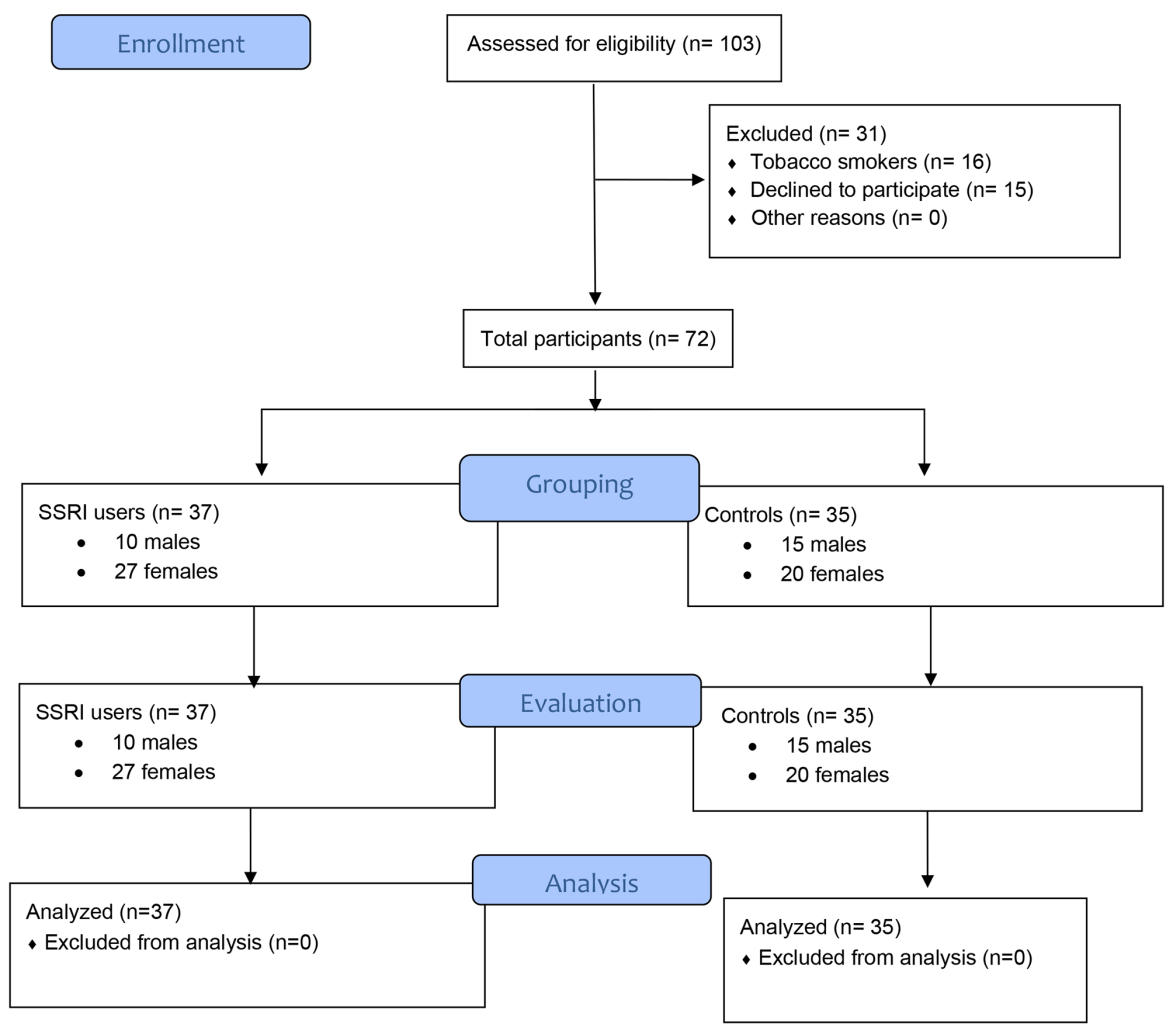
Flow diagram

One hundred and three individuals were invited. Thirty-one individuals were excluded. Among the excluded individuals 16 were current self-reported tobacco-smokers and 15 declined to sign the informed consent form. In total, 72 individuals, 37 SSRI users (10 males and 27 females) and 35 (15 males and 20 females) controls agreed to participate in the present study and reported to the healthcare facility for a clinical and radiographic evaluation (Fig. 1). Individuals using SSRI had a history of depression of 4.2 ± 2.5 years. The mean age of SSRI-users and controls were 48.7 ± 5.7 and 45.3 ± 5.1 years, respectively. A family history of mood disorders was more often reported by SSRI-users (51.4%) than controls (11.4%). All SSRI-users and 77.1% controls had attained university-level education. Tooth brushing 2x daily was reported by 75.7% and 62.9% SSRI-users and controls. Once daily flossing of interdental spaces was reported by 13.5% and 31.4% SSRI-users and controls, respectively. Users of SSRI and controls had their most recent visit to a dental facility 10.2 ± 0.6 and 7.5 ± 0.3 months ago, respectively (Table 1). All SSRI-users were diagnosed with mild depression and were prescribed Fluoxetine 20 mg/day as per medical records.

**Table 1:** Patient demographics

Parameters	Individuals using SSRI	Individuals not using SSRI
Number of participants	37	35
Male : Female	10 : 27	15 : 20
Mean age	48.7 ± 5.7 years	45.3 ± 5.1 years
Number of implants	53 implants	50 implants
Duration of implants in function	6.6 ± 0.7 years	7.04 ± 0.3 years
Duration of mood disorders	4.2 ± 2.5 years	NA
Family history of mood disorders	19 (51.4%)	4 (11.4%)
Highest level of education		
School level	NA	NA
College level	NA	8 (22.9%)
University level	37 (100%)	27 (77.1%)
Tooth brushing 2x daily	28 (75.7%)	22 (62.9%)
Flossing once daily	5 (13.5%)	11 (31.4%)
Most recent visit to oral health facility	10.2 ± 0.6 months ago	7.5 ± 0.3 months

NA: Not applicable

### Implants

A total of 53 and 50 single unit implants with similar dimensions were present in SSRI-users and controls, respectively. All implants were platform-switched with moderately rough surfaces and were placed in healed sites using insertion torques ranging between 30 and 35 N centimeters. Among SSRI-users and controls, 36 and 35 implants were located in the region of missing mandibular premolars and/or molars. All implants were restored with screw-retained prostheses and were in functions for 6.6 ± 0.7 and 7.04 ± 0.3 years in individuals using SSRI and controls, respectively (Table 2). None of the implants were placed in grafted sites.

**Table 2:** Dental implants

Parameters	Individuals using SSRI	Individuals not using SSRI
Number of implants	53 implants	50 implants
Duration of implants in function	6.6 ± 0.7 years	7.04 ± 0.3 years
Jaw location		
Anterior maxilla^*^	2	1
Anterior mandible^*^	—	—
Posterior maxilla^†^	15	14
Posterior mandible^†^	36	35
Insertion torque	30 to 35 Ncm	30 to 35 Ncm
Implant loading	Delayed	Delayed
Implant abutment connection	Platform-switched	Platform-switched
Implant dimensions		
Length	11 to 14 mm	11 to 14 mm
Diameter	4 to 5 mm	4 to 5 mm
Prosthesis retention	Screw-retention	Screw-retention

^*^Implants replacing missing incisors and/or canines

^†^Implants replacing missing premolars and/or molars

### Periodontal and peri-implant status

There was no statistically significant difference in PI, mPI, GU, mGI, PD, clinical AL, numbers of MT and mesial and distal MBL and CBL among individuals using SSRI compared with controls (Tables 3 and 4).

**Table 3:** Periodontal status

Parameters	Individuals using SSRI	Individuals not using SSRI
Plaque index	0.5 ± 0.05	0.3 ± 0.03
Gingival index	0.3 ± 0.03	0.2 ± 0.004
Probing depth	1.8 ± 0.2 mm	1.5 ± 0.08 mm
Clinical attachment loss	0.5 ± 0.08 mm	0.2 ± 0.04 mm
Marginal bone loss (mesial)	0.6 ± 0.1 mm	0.4 ± 0.05 mm
Marginal bone loss (distal)	0.8 ± 0.04 mm	0.5 ± 0.02 mm
Missing teeth	4.05 ± 0.6 teeth	3.8 ± 0.05

^*^Compared with individuals not using selective serotonin reuptake inhibitors (P < 0.05)

**Table 4:** Peri-implant soft tissue status and crestal bone loss

Parameters	Individuals using SSRI	Individuals not using SSRI
Modified plaque index	0.5 ± 0.03	0.2 ± 0.008
Modified gingival index	0.3 ± 0.05	0.2 ± 0.002
Probing depth	1.5 ± 0.2 mm	0.6 ± 0.04 mm
Crestal bone loss (mesial)	0.7 ± 0.08 mm	0.3 ± 0.005 mm
Crestal bone loss (distal)	0.5 ± 0.007 mm	0.3 ± 0.002 mm

### Saliva flow rate and interleukin 1-beta levels

The whole salivary flow rate in individuals using SSRI and controls was 0.11 ± 0.003 and 0.12 ± 0.001 ml/min, respectively. Whole salivary IL-1β levels in individuals using SSRI and controls were 57.6 ± 11.6 pg/ml and 34.6 ± 5.2 pg/ml, respectively.

### Correlation between demographic and periodontal and peri-implant parameters and whole salivary IL-1β levels

There was no statistically significant correlation between age, gender, duration since diagnosis of degression, periodontal and peri-implant clinicoradiographic parameters and whole salivary IL-1β levels in Users of SSRI and controls (data not shown).

## Discussion

The *null* hypothesis in the present study was that there is no difference in periodontal and peri-implant clinicoradiographic status and whole salivary IL-1β in participants using SSRI and controls (individuals not using SSRI). Results of the present investigation are in accordance with the hypothesis as no statistically significant difference in periodontal and peri-implant clinicoradiographic parameters and whole salivary IL-1β levels were observed in users of SSRI and controls. Therefore, it is alluring to conclude that use of SSRI does not negatively affect periodontal and peri-implant soft and osseous tissues. However, such a conclusion should be cautiously interpreted as our results were in contradiction to previous studies [6, 7, 17–21]. There are a number of factors that may have influenced the results reported in the present investigation. An underprivileged education status (UES) is a significant risk-factors of oral diseases including periodontitis [32, 38]. In a recent cross-sectional study on 296 individuals Kareem et al. [3] showed that periodontitis is more often manifested in users of SSRI than controls. This study [3] also demonstrated a statistically significant correlation between female gender, UES and periodontitis in users of SSRI. Similarly, in the study by Bey et al. [17] scores of GI, clinical AL, and PD are higher in users of SSRI than controls. It is noteworthy that in the present investigation users of SSRI were well-educated in general as at least 75% individuals reported to have attained University-level education. Moreover, toothbrushing twice daily and flossing of interproximal spaces was reported by nearly 76% and 14% of SSRI-users. Moreover, SSRI-users and controls reported to have visited an oral healthcare provider within a year. Since all SSRI-users had mild depression, it is likely that these individuals were conscious of their health including oral health and were taking measures to maintain a healthy life style. In contrast, in the study by Bey et al. [17] participants had moderate to severe depression. Although level of patient education was not assessed by Bey et al. [17] it is speculated SSRI-users in this study had an UES. Therefore, an UES accompanied with more severe depression could have forced participants to disregard routine health activities such as OHM and also neglect intake of medications including SSRI. It is pertinent to mention that assessment of severity of psychological disorders including depression is a critical factor that may potentially impact the severity of periodontal and peri-implant inflammatory conditions. It is also suggested that evaluation of depression should be assessed in “self-reported medically healthy individuals” to determine presence of such disorders in potentially undiagnosed individuals. The authors perceive that a clear understanding of participants’ medical history and routine communication with participants’ psychiatrist/psychologist/ medical doctors plays an important role in assessing the participants’ oral health care needs. This may also positively influence outcomes of oral interventions such as periodontal and peri-implant therapy and the overall quality of life of individuals especially those with psychiatric and/or mood disorders.

The IL-1β is a proinflammatory cytokine that is expressed in raised concentrations in the UWS of participants with periodontitis and peri-implant diseases [39, 40]. An increased production of IL-1β has been reported to enhance osteoclastic activity [41, 42]. The present results showed no significant difference in whole salivary IL-1β levels in SSRI-users and controls. Moreover, all participants (SSRI-users as well as non-users) demonstrated mesial and distal MBL and CBL of approximately 2 mm, which does not indicate periodontitis or peri-implantitis, respectively. Results from studies [13, 15, 43] on rats have shown that fluoxetine exerts anti-inflammatory effects and favors bone formation. From the present results, it is demanding to solely credit Fluoxetine in minimizing whole salivary IL-1β levels, periodontal and peri-implant inflammation and the risk of MBL and CBL in SSRI-users. Since all participants had adequate plaque control with no periodontal pockets of 4 mm or greater, the importance of routine OHM in this regard cannot be discredited.

Cortisol is a hormone produced by adrenal glands [44–46]; and episodes of psychological stress have been reported to elevate cortisol levels (CL) in bodily fluids including UWS, serum and peri-implant sulcular fluid [47]. In this context, CL are often measured to screen participants with psychiatric disorders including depression [48, 49]. It is noteworthy that CL were not assessed in the present investigation. Although this may be labelled as a limitation of the present investigation, it is pertinent to mention that a diagnosis of diagnosis of depression was pre-established via evaluation of participants’ medical records during the initial patient screening process. However, it is anticipated that whole salivary CL, if measured, would have been comparable and within normal limits among participants with and without depression included in the present investigation. Once again, this could be associated with the duration and severity of depression (mild depression) and satisfactory oral hygiene status of patient population. Moreover, there is a possibility that use of SSRI plays a role in modulating whole salivary CL in modulating inflammation as well as CL in participants with depressive symptoms. It is hypothesized that whole salivary CL are higher in participants with severe depression. Another limitation is that the design of the present study was purely observational. Since none of the SSRI-users and controls were diagnosed with periodontitis, it is speculated that traditional periodontal treatments such as non-surgical mechanical debridement (MD) of periodontal and peri-implant sulci and implant/tooth surfaces is sufficient to treat periodontal soft tissue inflammation in these participants. However, in SSRI-users and controls with severe depression and periodontal and peri-implant diseases, interventions such as photobiomodulation and photodynamic therapy as adjuncts to MD may be warranted.

## Conclusion

Users of SSRI and controls demonstrate healthy periodontal and peri-implant tissue statuses with no marked differences in whole salivary IL-1β levels provided oral hygiene is stringently maintained.

## Data Availability

The datasets that were used in the present study are available from the corresponding author on reasonable request. The data are not publicly available due to data protection guidelines according to the ethics approval.
